# Genetic variation in the *NEIL2* DNA glycosylase gene is associated with oxidative DNA damage in *BRCA2* mutation carriers

**DOI:** 10.18632/oncotarget.22638

**Published:** 2017-11-23

**Authors:** Carlos Benítez-Buelga, Juan Miguel Baquero, Tereza Vaclova, Victoria Fernández, Paloma Martín, Lucia Inglada-Perez, Miguel Urioste, Ana Osorio, Javier Benítez

**Affiliations:** ^1^ Human Genetics Group, Spanish National Cancer Research Center (CNIO), Madrid, Spain; ^2^ Endocrine Cancer Group, Spanish National Cancer Research Center (CNIO), Madrid, Spain; ^3^ Familial Cancer Unit, Spanish National Cancer Research Center (CNIO), Madrid, Spain; ^4^ Spanish Network on Rare Diseases (CIBERER), Madrid, Spain

**Keywords:** BRCA1 and BRCA2, NEIL2 polymorphism cancer risk modifier, mRNA levels, oxidative DNA damage

## Abstract

In this report, we have tried to gain molecular insight into a single nucleotide polymorphism (SNP) in the *NEIL2* gene previously identified as “cancer risk modifier” for *BRCA2* mutation carriers.

To that end, we studied the role of this SNP (rs804271) on *NEIL2* transcriptional regulation, oxidative DNA damage and genome instability in two independent set of samples: The first one was a series of eighty-six *BRCA1* and *BRCA2* mutation carriers and eighty non-carrier controls in which we evaluated the effect of the SNP on *NEIL2* gene expression and oxidative DNA damage accumulation. The second was a set of twenty lymphoblastoid cell lines (LCLs), thirteen *BRCA1* mutation carriers and seven non-carriers control, that were used to analyze the correlation between *NEIL2* mRNA and/or protein levels, the oxidative and the double stranded break (DSB) DNA damage levels.

Our results suggest that an excessive production of NEIL2 enzyme, associated with the SNP, may have a deleterious effect modifying cancer risk susceptibility in *BRCA2* mutation carriers. We hypothesize that due to the SNP impact on *NEIL2* transcriptional upregulation, a cascade of events may converge in the accumulation of oxidative DNA damage and its posterior conversion into DSBs for this specific group of patients.

## INTRODUCTION

The tumor suppressor genes *BRCA1* and *BRCA2* maintain genomic stability through their involvement in homologous recombination (HR) double-stranded break DNA repair among other processes [1].

Carrying a mutation in the *BRCA1* or *BRCA2* genes increases a woman’s lifetime risk of developing breast and ovarian cancer, although there are considerable differences in disease manifestation. At the age of 80, cumulative cancer risk for *BRCA1* and *BRCA2* mutation carriers ranges from 72% to 69% for breast cancer development, and from 44% to 17% for ovarian cancer [2]. This high variability may be explained by other genetic modifiers and/or environmental factors.

Given the relation of synthetic lethality that exists between one of the components of the Base Excision Repair (BER) pathway, *PARP1* (poly[ADP-ribose] polymerase 1), and both *BRCA1* and *BRCA2* genes [3], it is likely that other members of the BER pathway exhibit a similar behavior. We hypothesized that common genetic variants in genes involved in BER might modify a woman’s lifetime risk of developing breast and ovarian cancer if she is a *BRCA1* or *BRCA2* mutation carrier. In particular, two Single Nucleotide Polymorphisms (SNPs) in the *OGG1* and *NEIL2* genes were identified as cancer risk modifiers for *BRCA1* and *BRCA2* mutation carriers, respectively [4]. Although the molecular mechanism underlying these associations is not clear yet, both SNP_S_ were in transcriptional regulatory regions of genes encoding DNA glycosylase enzymes which play an important role in the first steps of the pathway.

The BER pathway corrects base lesions from deamination, oxidation or methylation [5, 6] which represent the majority of endogenous DNA damage due to chemical reactions during cellular metabolism [7]. There are 11 DNA glycosylases which have the ability of recognizing a wide variety of lesions thanks to a DNA binding domain, the helix-hairpin helix DNA binding motif (like OGG1) [8] and the helix-2turn-helix domain (like NEIL2) [9]. In bi-functional DNA glycosylases, like OGG1 or NEIL2, base lesions are excised from the DNA thanks to its glycosylase activity and AP lyase activity, although they may have different DNA-structure/substrate affinities. For example, the OGG1 incises DNA at 8-oxoG residues, and is active only on duplex DNAs [10]. In contrast, NEIL2 shows preferential activity on bubble DNA or single-stranded DNA regions [11] and present high incising activity for several cytosine-derived lesions with robust activity for 5-hydroxyuracil and weaker activity for dihydrouracil, 5-hydroxycytosine, thymine glycol and 8-oxoG [10].

If they are not repaired, these lesions may evolve into mutation (C:G→T transversions [12] or DNA single-strand [7] or double-strand breaks (DSBs) [13, 14], which are the principal source of genomic instability [15, 16].

Certain SNPs in DNA glycosylase genes could affect negatively to the general performance of the BER pathway and contribute by increasing the levels of genome instability and hence to a higher cancer risk, especially in presence of a defective *BRCA1* or *BRCA2* background. As an example, we previously identified that the single nucleotide polimorphism “rs2304277”, located 1.8Kb downstream the 3′-untranslated region (UTR) of *OGG1* gene, was associated with an increased ovarian cancer risk for *BRCA1* mutation carriers [4]. We tried to explain this cancer association at a molecular level and we discovered that the SNP was associated with a constitutive *OGG1* transcriptional down-regulation, which contributed to a higher genome and telomere instability, especially in those individuals harboring mutations in *BRCA1* [17].

Similarly, the SNP rs804271, localized within the *NEIL2* promoter region, is associated with increased breast cancer risk for *BRCA2* mutation carriers [4]. This SNP forms part of several transcription-factor binding motifs that are responsive to oxidative stress [18]. It has previously been reported that SNPs 5´- UTR upstream the coding region of the *NEIL2* gene influence gene transcription levels and alter levels of genetic damage [19]. In this study, we have explored in two independent set of samples with different BRCA status the role of this SNP at transcriptional level and its possible implication on DNA damage and genome instability to explain its cancer risk modifier effect.

## RESULTS

### SNP frequency in FBOC series

We genotyped the rs804271 in FBOC (familial breast and ovarian cancer) individuals, and we found a SNP allelic frequency of 0.39, similar as reported for European population 0.41 in Ensembl data base (http://www.ensembl.org). No significant differences in the genotypic frequencies were detected among the different BRCA and control groups (Supplementary Table 1).

### *NEIL2* mRNA levels are activated by rs804271 SNP: *In silico* studies (HaploReg and GTEX public data), FBOC series and LCLs

The SNP rs804271 is located at the 5′- UTR region of the *NEIL2* gene, within a transcriptional regulatory domain at Transcriptional Start Site (TSS) of the gene. We explored the possible phenotypic effects of this SNP by using HaploReg Database web server [20] and we found that 18 proteins are predicted to interact within TSS and 3 binding motifs for transcription factors TFs (E2F1, SIN3A and YY1) are predicted to be altered in the presence of this specific SNP (rs804271), (Supplementary Table 2).

Because transcriptional changes could be expected due to the modifications by this SNP at the TSS, we used the GTEx eQTL web server [21] (http://www.gtexportal.org) to test whether rs804271 was associated with changes on *NEIL2* mRNA levels in different tissues. Overall, we found significant increased *NEIL2* mRNA levels for 30 tissues, including breast (*p* = 1*10^−4^), ovary (*p* = 1.4 * 10^−14^), and blood (*p* = 6.6 * 10^−13^), Supplementary Table 3 although in some of them, such as “Cells - EBV-transformed lymphocytes (LCLs)” the effect was “moderated” (Supplementary Figure 1).

**Figure 1:**
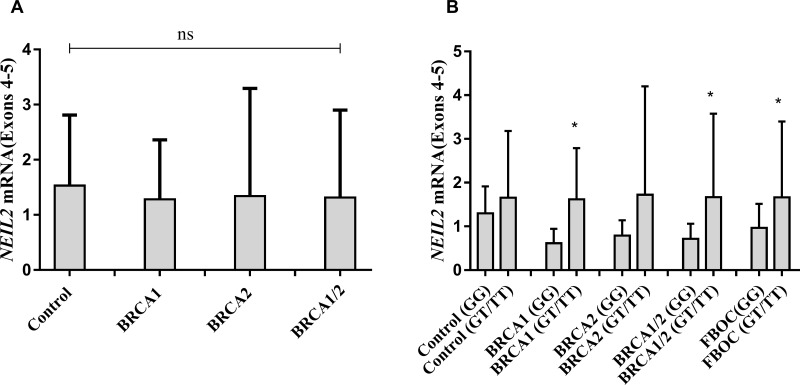
(**A**) Comparative analysis of *NEIL2* mRNA expression according BRCA mutational status in FBOC series (*BRCA1* and *BRCA2* mutation carriers are compared with Controls). (**B**) Comparative analysis of *NEIL2* mRNA expression according the SNP status ((Carriers (GT/TT) *Vs* Non-carriers (GG)) among the different FBOC groups (*BRCA1, BRCA2* mutation carriers and *BRCA1/BRCA2* non-carrier Controls). Bars represent the mean and the standard deviation for each group. Unpaired student t test was used to test for potential significant differences between means. (*p < 0.05).

In parallel, we measured *NEIL2* mRNA expression levels in FBOC series considering both, the BRCA mutational status and the presence or absence of the NEIL2- variant to stratify and compare expression values among groups. We found no significant differences in the *NEIL2* mRNA levels between *BRCA1* and/or *BRCA2* mutation carriers compared to controls (Figure 1A). In contrast, when we stratified by the presence of the SNP we detected a common *NEIL2* mRNA up-regulation pattern that was similar for each BRCA mutational group (Figure 1B). We performed linear regression analysis to confirm that the rs804271 was associated with significant higher *NEIL2* mRNA levels (β = 0.24; *p* = 0.01) among the FBOC individuals.

Finally, we measured *NEIL2* mRNA basal levels among the 20 LCLs considering the BRCA and SNP status. Although we detected higher *NEIL2* mRNA levels for those LCLs harboring the SNP, these differences were not significant (Supplementary Figure 2). This result confirmed the tissue variability previously observed in the data provided by GTEX (Supplementary Figure 1).

### NEIL2 mRNA and protein levels are correlated and both predict NEIL2-derived DNA damage

**Figure 2:**
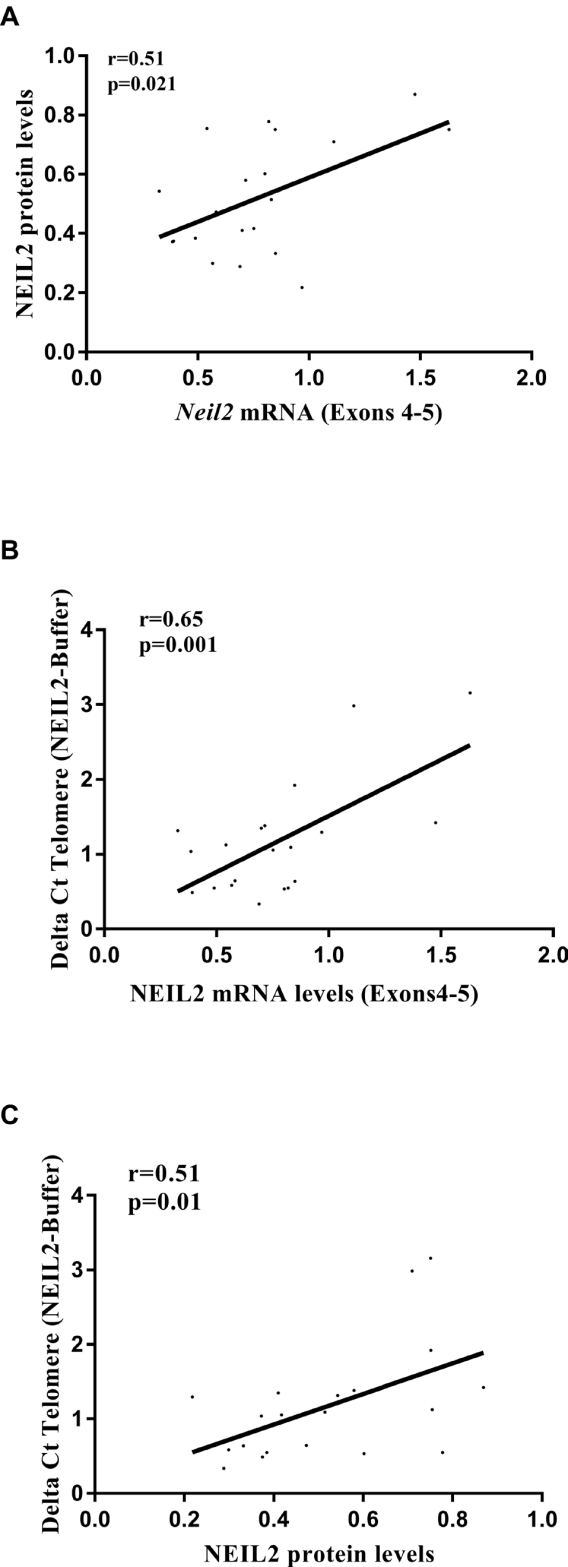
(**A**) Correlation analysis between *NEIL2* mRNA and protein levels. (**B**) Correlation analysis between *NEIL2* mRNA levels and the relative amount of “NEIL2-lesions”. (**C**) Correlation analysis between the NEIL2 protein levels and the relative amount of “NEIL2-lesions”. Spearman test, was used to test whether correlation is significant. significant *p*-value when (*p* < 0.05).

Because protein sample from FBOC series was not available, we decided to use the LCL panel (*n* = 20) to test NEIL2 protein levels (Supplementary Figure 3). Spearman correlation analysis confirmed that *NEIL2* mRNA and protein levels were significantly correlated among LCLs in basal conditions (*r* = 0.51; *p* = 0.02), Figure 2A.

In parallel, we measured in the DNA extracted from the same LCLs (*n* = 20), the amount of base lesions that are recognized and processed by NEIL2 (NEIL2-lesions) at telomeres (detailed information in the material and methods section). We selected this region because NEIL-protein family members have been described to be active at telomeres [22]. Then, we performed a correlation analysis between the *NEIL2* mRNA/ protein levels and the relative number of “NEIL2-lesions” detected, independently of the BRCA or the SNP status. We found that both *NEIL2* mRNA and NEIL2 protein levels were significantly correlated with the relative number of telomeric “NEIL2-lesions¨ (*r* = 0.65; *p* = 0.001 and *r* = 0.51; *p* = 0.01, respectively), (Figure 2B and 2C).

### The rs804271 is associated to higher levels of NEIL2-lesions at telomeres in FBOC series

**Figure 3:**
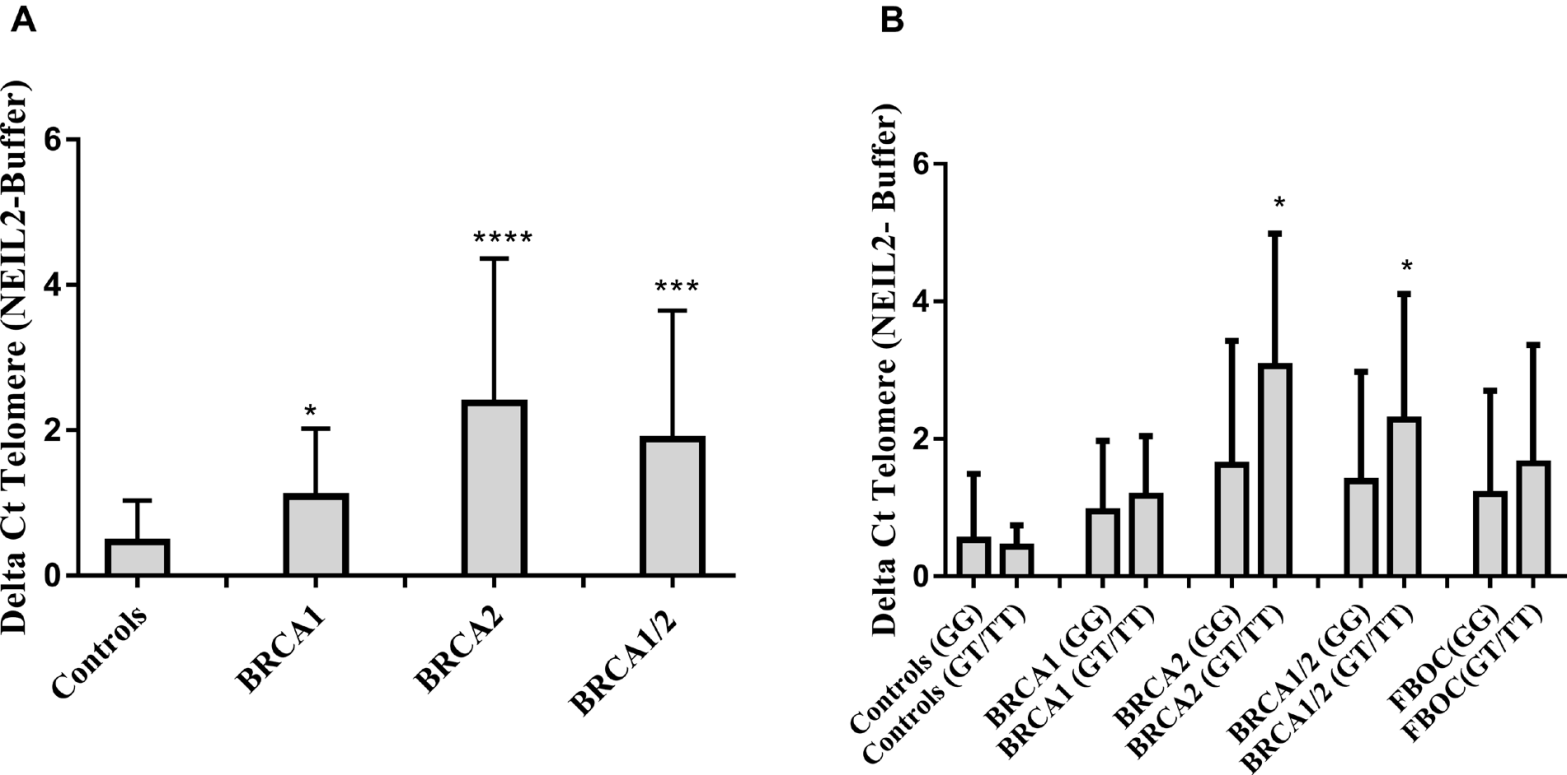
(**A**) Comparative analysis of the relative number of NEIL2-lesions found at telomeres according BRCA mutational status in FBOC series (*BRCA1* and *BRCA2* mutation carriers are compared with Controls). (**B**) Comparative analysis of the relative amount of “NEIL2-lesions” found at telomeres according the SNP status ((Carriers (GT/TT) *Vs* Non-carriers (GG)) among the different BRCA mutational groups in FBOC series (*BRCA1, BRCA2* mutation carriers and *BRCA1/BRCA2* non-carrier Controls). Unpaired student *t* test was used to test for potential significant differences. (*p < 0.05, **p < 0.01, ***p < 0.001, ****p < 0.0001).

When considering the BRCA status and NEIL2 genotypes, we found significantly higher amount of “NEIL2-lesions” in *BRCA1* and *BRCA2* mutation carriers compared with controls (*p* = 0.01 and *p* < 0.0001 respectively), (Figure 3A). Moreover, when we considered the presence of the SNP (rs804271) we found that those individuals presenting both genetic events (BRCA mutation together with the SNP) presented significantly higher levels of “NEIL2-lesions”, compared to their *BRCA1/BRCA2* counterparts without the SNP or controls (*p* < 0.05), (Figure 3B).

Because FPG (formamidopyrimidine [fapy]-DNA glycosylase) (e. *coli*) recognizes specifically oxidative purines lesions (8-oxoG/methylFapyG) [23], we measured in the DNA from our FBOC individuals the relative amount of “FPG-lesions”. Then, we performed correlation analysis between (“FPG-lesions” and “NEIL2-lesions”) and we detected a significant correlation between both type of lesions (*r* = 0.40; *p* = 0,03), (Supplementary Figure 4A), which suggest that that from the wide range of lesions that NEIL2 can recognize [10], the presence of the SNP among *BRCA1* and *BRCA2* mutation carriers lead preferentially to the accumulation of purine lesions (8-oxoG or methylFapyG).

Because telomeres are susceptible to uracil miss incorporation which is primarily recognized and removed by the uracil DNA glycosylase (UNG) [24], we have measured the relative amount of uracil miss incorporation at the telomere region as NEIL2 is not able to recognize/process this type of lesions. We performed correlation analysis between “Uracil-lesions” and “NEIL2-lesions” and we found no significant correlation between them (Supplementary Figure 4B)

### NEIL2-derived DNA damage correlates with γH2AX intensity signal

**Figure 4:**
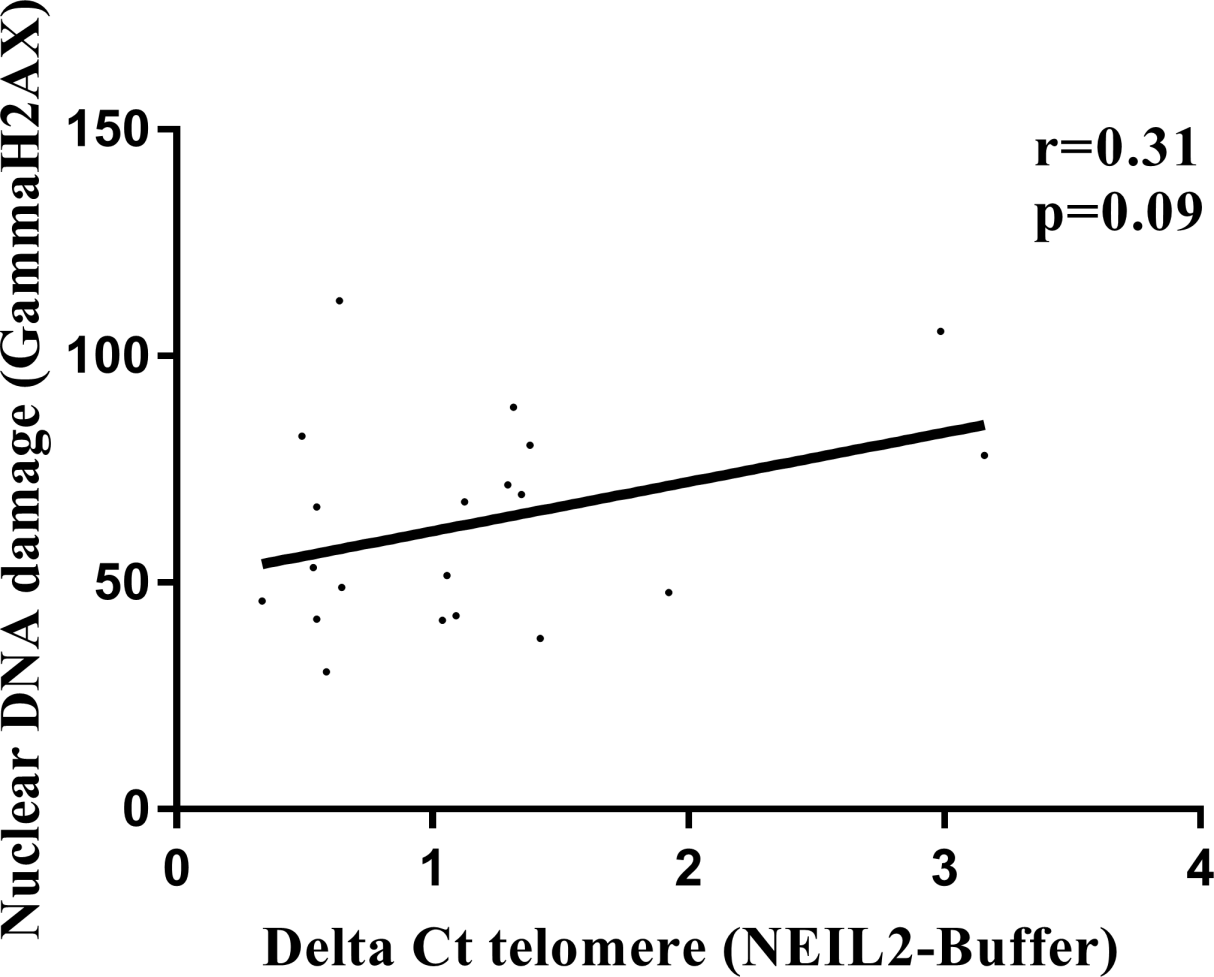
Correlation analysis between relative amount of “NEIL2-lesions” and the γH2AX nuclear intensity signal (DSBs) Spearman test, was used to test whether correlation is significant. significant *p*-value when (*p* < 0.05).

We measured the γH2AX signal intensity in the cell nucleus of the 20 LCLs (as a marker of DSBs) at basal conditions. We found a direct correlation between the relative amount of “NEIL2-lesions¨ and the nuclear γH2AX intensity signal independently of the BRCA or SNP status (*r* = 0.31; *p* = 0.09), (Figure 4).

## DISCUSSION

In the present study, we have tried to gain molecular insights into a common genetic variant (rs804271) previously reported by our group to be associated with increased breast cancer risk in *BRCA2* mutation carriers [4]. For that, we have used two independent set of samples to test the SNP effect on *NEIL2* transcriptional regulation and its possible implication on genome instability.

This SNP is localized within the TSS of *NEIL2* gene. Previous characterization of the *NEIL2* promoter region showed that *NEIL2* transcription is influenced by certain SNPs located 5′ upstream of the start site [19]. Indeed, *in silico* analysis predicted that this polymorphism is located within a binding motif for several transcription factors (Supplementary Table 2), and transcriptional modifications due to this SNP may be expected.

Data from Gtex confirmed that the presence of rs804271 was associated with a significant mRNA upregulation in 30 tissues including breast (*p* = 0.00001), ovary (*p* = 1.4 * 10^−14^), and blood (*p* = 6.6 * 10^−13^), (Supplementary Table 3). However, for some tissues, such as “Cells - EBV-transformed lymphocytes (LCLs)”, this effect was “moderate” (Supplementary Figure 1), suggesting that the intensity of the SNP effect may be tissue specific. We validated these results in our FBOC series and we found, independently of the BRCA status, significantly increased *NEIL2* mRNA levels in the blood from FBOC individuals harboring the SNP (β = 0.24; *p* = 0.01), suggesting that it is associated *per se* with transcriptional activation of the *NEIL2* gene. In contrast, we were not able to detect a significant *NEIL2* mRNA upregulation associated to the SNP in the 20 LCL analyzed, confirming the tissue specificity found in the GTEX data. All these results suggest that rs804271 is indeed associated with constitutive transcriptional activation of the *NEIL2* gene.

A recent work in which NEIL1 and NEIL2 (Neil1 −/− /Neil2 −/−) double and NEIL1, NEIL2 and NEIL3 (Neil1 −/− /Neil2 −/− /Neil3 −/−) triple knock-out mouse models have been characterized, no accumulation of oxidative DNA damage, no changes in the mutation frequencies under normal physiological conditions and more importantly, no cancer predisposition for these mice has been observed [25]. This would agree with our results in which it is *NEIL2* “excess” and not its “absence” that may be deleterious and responsible for the increased risk effect of this SNP in *BRCA2* mutation carriers.

In the line of this hypothesis, it has been previously described that *NEIL2* gene is frequently amplified in esophageal adenocarcinoma and that tumors with copy number gains of *NEIL2* gene present significant poor prognosis [26, 27]. In addition, we have observed that *NEIL2* gene is frequently upregulated in several tumor types (Supplementary Figure 5A), and more importantly that *NEIL2* mRNA upregulation or copy number amplification has prognostic value for some of those tumors (Supplementary Figure 5B).

The molecular mechanism by which *NEIL2* mRNA upregulation could be deleterious for *BRCA2* mutation carriers is unclear. However, high expression levels of BER related enzymes have been associated with tissue oxidative DNA base damage [12]. In addition, it was described that rs804271 (previously ss74800505) was associated with both NEIL2 transcriptional modifications and significantly increased mutagen-induced genetic damage [19]. In fact, in LCLs we found a significant positive correlation between the amount of *NEIL2* mRNA or protein levels and “NEIL2-lesions” (*r* = 0.65; *p* = 0.001 and *r* = 0.51; *p* = 0.02, respectively) (Figure 2B). Moreover, in FBOC the SNP was also associated with higher amount of “NEIL2-lesions” compared to their counterparts without the SNP, although it was only significant for *BRCA1* and *BRCA2* mutation carriers (*p* = 0.03) (Figure 3B).

A possible explanation for this result, could be that the NEIL2 enzyme “excess” as consequence of the SNP could lead to the recognition and binding to DNA lesions for which normally it presents low excision activity, like 8-oxoG [10]. Indeed, we found a significant correlation between “NEIL2-lesions” and “FPG-lesions” (*r* = 0.40; *p* = 0.003) (Supplementary Figure 4A), which mostly correspond to purine bases lesions (8-oxoG/methylFapyG). This could lead to a delay in the repair and to the accumulation of “NEIL2-lesions” in the DNA.

In the context of *BRCA1* and *BRCA2* deficiency, this accumulation of base lesions would be deleterious since both enzymes are involved in transcription-coupled repair of 8-OxoG [28] and protect against oxidative DNA damage converted into DSBs [14]. Indeed, our results in the LCLs confirmed that the relative number of “NEIL2-lesions” at the telomere was correlated with nuclear γH2AX intensity signal (a marker for DSBs) independently of the BRCA or SNP status (*r* = 0.31; *p* = 0.09) (Figure 4).

In summary, our hypothesis would be that this SNP activates at transcriptional level *NEIL2* gene expression leading to a cascade of events that converge in the accumulation of unresolved “NEIL2-lesions” that may be converted into DSBs. In a system with a defective HR DNA repair, as it is the case for *BRCA2* mutation carriers, this SNP would contribute to higher genome instability and finally to a higher cancer risk for this specific group of patients.

## MATERIALS AND METHODS

### Familial breast and ovarian cancer (FBOC) series

We studied a group composed of 166 individuals belonging to 51 families meeting high-risk criteria, and screened for deleterious mutations in the *BRCA1* and *BRCA2* genes, as reported previously [29]. Of these families, 25 carried a deleterious mutation in the *BRCA1* gene, 25 in *BRCA2*.

Eighty individuals were used as non-carrier controls: they were relatives of *BRCA1/2* mutation carriers who did not have personal cancer antecedents and did not harbor the corresponding familial mutation in the *BRCA1* or *BRCA2* genes.

All cases and controls signed an appropriate informed consent form and the ethics committee of the hospital involved (Fuenlabrada University Hospital) approved the proposal.

We used this set of samples to calculate the SNP frequency, to quantify *NEIL2* mRNA levels in peripheral blood and to measure the accumulation of oxidative DNA damage at telomeres (NEIL2-lesions, FPG-lesions, uracil accumulation) in blood DNA. (Table 1).

**Table 1:** FBOC series information

	Families (n)	Healthy carriers (n)		Cancer cases (n)	rs804271 genotyped (n)	NEIL2 mRNA (n)	NEIL2-lesions	FPG-lesions	uracil-lesions
***BRCA1***		25	21	19	40	24	25	14	14
***BRCA2***		25	23	23	46	30	35	18	18
***Controls***		na	0	0	80	29	25	20	20

Information regarding number of healthy *BRCA1* and *BRCA2* mutation carriers or cancer cases and the sample size for each experimental section.

### Lymphoblastoid cell lines

A second set of 20 LCLs was established by Epstein-Barr virus transformation of peripheral blood lymphocytes from thirteen healthy women carrying heterozygous mutations in *BRCA1* and seven non-carrier relatives used as controls. Mutational analysis had been performed by Sanger sequencing (BRCA status) or Taqman probe (rs804271) (Supplementary Table 4). None of the women included in the study had personal antecedents of cancer. This LCL panel has been previously described by our group [30]. Cell lines were cultured in RPMI-1640 media (Sigma-Aldrich) supplemented with non-heat-inactivated 20% fetal bovine serum (Sigma-Aldrich), penicillin-streptomycin (Gibco) and Fungizone (Gibco). The cultures were carried out in 25 cm^2^ flasks (Corning) at 37°C in 5% CO2 atmosphere and cell lines were maintained in exponential growth by daily dilution to 106 cells/ml of full media.

We used this sample set to analyze the correlation between *NEIL2* mRNA – protein levels, the relative number of “NEIL2-lesions” found at DNA, and the relative number of double stranded brakes (DSB) at DNA.

### SNP genotyping (rs804271)

Single Nucleotide Polymorphism rs1466785, located in the *NEIL2* gene is a cancer risk modifier for *BRCA2* mutation carriers [4]. Imputation using *1000 Genomes* data showed that there were several SNPs in strong linkage disequilibrium (LD) with rs1466785, the original SNP reported in Osorio *et al.* [4]. Of these, we considered rs804271 to be the best candidate, given that it showed the most significant associations and that there existed functional data supporting its putative role in cancer [19].

DNA was extracted from peripheral blood of FBOC patients or LCLs using MagNAPure LC 2.0 (Roche Diagnostics, Indianapolis, Indiana) following the manufacturer’s instructions. DNA quantification and quality were assessed by NanoDrop^®^ (ND-1000 V3.7.1). A specific Taqman probe for rs804271 was used to genotype the presence/absence of the polymorphism among the sample collection. Allelic discrimination assays were conducted using the 7900HT Fast Real-Time PCR System (Applied Biosystems). Probe design for rs804271 is (G>T) instead of (C>A). Along the manuscript we refer to the variant as G>T.

### *NEIL2* mRNA expression analysis

RNA was extracted from peripheral blood cells using TRIzol Reagent (Ambion^®^, Life Techonogies) according to the manufacturer’s instructions. NanoDrop^®^ (ND-1000 V3.7.1) was used to assess both RNA quantity and quality. Two microliters of cDNA at a final concentration of 10-20 ng/μl were mixed in triplicate with GoTaq^®^ qPCR MasterMix 1x (Promega), *NEIL2* cDNA primers (F/R) and GAPDH cDNA primers (F/R) at final concentrations of 500nM. Primers used were: *NEIL2 4-5 exons* (F: GTCACACCCACCTGTGACAT; R: GCACTCAGGACTGAACCGAG) and *GAPDH* (F: CCTGCACCACCAACTGCTTA; R: CCATCACGCCACAGTTTCC).All reagents were used following the manufacturer’s instructions. qPCR was done using the QuantStudio S6 system (Applied Biosystems).

### *NEIL2* protein quantification

The expression level of endogenous NEIL2 protein was analyzed by western blot. Briefly, cell lysates were prepared in RIPA buffer (Sigma) and protease inhibitors cocktail (Roche). Protein content was determined by Lowry analysis (Bio-Rad). Eighty micrograms of proteins were analyzed by SDS-PAGE on polyacrylamide gels and transferred to Immobilon-FL membranes (Millipore). Membranes were blocked in TBS-T (50 mM Tris-HCl, 150 mM NaCl, pH 7.5 plus 0.1% Tween 20) and 5% nonfat milk for 1 hour at RT. Blots were probed with following primary antibodies: rabbit anti-NEIL2 (Atlas Antibibodies, #HPA064460) at 1/1000 dilution or mouse anti-GAPDH (manufactured by the monoclonal antibodies core nit from the Spanish National Cancer Research Centre) at 1/3000 dilution in TBS-T containing 5% nonfat milk. The secondary antibodies were HRP-conjugated (Dako) and the immunoblots were developed using the ECL system (GE Healthcare). ImageLab software version 4.1 (Bio-Rad) was used for image acquisition and images were analyzed using ImageJ software for quantification of signal intensity/area for both proteins.

### Oxidative DNA damage studies “NEIL2-lesions”

We used a qPCR-based method to evaluate the oxidative DNA damage within telomeric DNA [32], based on differences in PCR kinetics between DNA template digested by formamidopyrimidine-DNA glycosylase (FPG) and undigested DNA. Quantitative real-time amplification of genomic DNA was performed as described by O’Callaghan et al. [31].

### Measurement of telomere damage

#### Oxidative DNA damage within telomeres

We used a qPCR-based method to evaluate the oxidative stress within telomeric DNA. We followed the procedure described by O’Callaghan *et al.* based on differences in PCR kinetics between DNA template digested by formamidopyrimidine-DNA-glycosylase (FPG) and undigested DNA [32]. Briefly, FPG is a bacterial DNA glycosylase that recognizes and cuts the oxidized bases from DNA, principally 8-oxoG, AP sites that are converted in single-strand breaks (SSBs) by its AP-lyase activity. These SSBs reduce amplification efficiency, thus, the ΔCq after digesting DNA by FPG (Cq digested – Cq undigested) is proportional to the oxidative damage in the amplified region. The incubation and qPCR amplification of genomic DNA was performed as described by O’Callaghan et al. [31].

### Quantification of “NEIL2-lesions” accumulation at telomeres

The telomere oxidation protocol previously described can be potentially adapted to quantify the accumulation of different base lesions incubating the DNA with other glycosylases that are sensitive to other specific base lesions. Following this premise, we used NEIL2 enzyme to measure the “NEIL2-lesions” accumulation (5hydroxyuracildihydrouracil, 5-hydroxycytosine, thymine glycol and 8-oxoG) at telomeres [9]. We optimized the protocol using a low NEIL2 concentration, decreasing DNA amount and incubation time. 200 ng of genomic DNA was incubated with 5,6 μM NEIL2 (provided by Dr. Thomas Helleday, Karolinska Institutet, Stockholm, Sweden) or without (replaced with H20) in a buffer (25 mM TrisHcl pH 8.0, 15 mM NaCl, 2 Mm MgCl2 and 0.0025% Tween 20) for 4 hours at 37°C. The reaction was stopped by incubation at 95°C for 5 min. qPCR analysis was performed on 10 ng of digested or undigested genomic DNA following the same reagents and conditions that in the original protocol for FPG [31].

### Quantification of uracil accumulation at telomeres

Following this premise, we used UNG to measure the accumulation of uracil at telomeres that is recognized and excised by this enzyme [33]. We optimized the protocol using a low UNG concentration, decreasing DNA amount and incubation time. 180 ng of genomic DNA was incubated with 130 nM UNG (provided by Dr. Thomas Helleday, Karolinska Institutet, Stockholm, Sweden) or without (replaced with H20) in a buffer (25 mM TrisHcl pH 8.0, 15 mM NaCl, 2 Mm MgCl2 and 0.0025% Tween 20) for 30 min at 37°C. The reaction was stopped by incubation at 95°C for 5 min. qPCR analysis was performed on 10 ng of digested or undigested genomic DNA following the same reagents and conditions that in the original protocol for FPG [31].

### DNA damage

LCLs were cultured 4 hours before fixation with 4% paraformaldehyde (Electron Microscopy Sciences, Hatfield, Philadelphia, USA). Two hours before fixation, cells were counted and seeded into a poly-L-lysine-coated (Sigma-Aldrich) μCLEAR bottom 96-well plate (Greiner Bio-One) at a density of 75,000 cells per 100ul full media per well. LCL were then left for 2 hours to attach to the surface of the wells, fixed for 15 min at room temperature, permeabilized in 0.5% Triton X-100 in PBS for 20 minutes at 4°C and stained with primary and secondary antibodies and 4′,6-Diamidino-2-phenylindole dihydrochloride (DAPI) to visualize nuclei. To detect γ-H2AX we used mouse monoclonal anti-phospho-histone H2AX antibody (Millipore; #05-636). Alexa Fluor 488 from molecular probes (Invitrogen; #A-11034) was used, and fluorescent images were automatically taken for each well of the 96-well plate using an Opera High-Content Screening System (Perkin Elmer). Pictures were taken under non-saturating conditions using a 40x magnification lens to calculate the γ-H2AX nuclear signal intensity.

### Statistical analysis

Pearson’s chi-squared test was used to calculate whether differences in the frequency of the SNP among the FBOC groups were significant (Supplementary Table 1).

We performed linear regression analysis to test whether cancer antecedents in *BRCA1* and *BRCA2* mutation carriers were associated with any of the variables we evaluated in this report, but we did not find significant differences (Significant *p*-values < 0.05) between healthy *BRCA1* and *BRCA2* carriers or cancer cases. Hence, we did not stratify for cancer status in these groups (Supplementary Table 5).

We considered heterozygotes and homozygotes (GT/TT) as a single group, to evaluate the effect of the SNP for each of the studied variables, as the cancer modifier effect of rs804271 is dominant for *BRCA2* mutation carriers [4].

Significant differences for the different comparative analysis were stablished by unpaired *t* test analysis (SNP effect on *NEIL2* mRNA levels or NEIL2 derived base damage accumulation, Figure 1 and Figure 3, respectively).

Spearman correlation was used to assess for significant correlations between *NEIL2* mRNA levels, protein levels and NEIL2 derived base damage accumulation at telomeres (Figure 3). Also, to assess whether NEIL2-lesions correlates with “FPG-lesions”, “UNG-lesions” and γ-H2AX nuclear signal intensity in FBOC and LCLs respectively (Supplementary Figure 4A and Figure 4B).

Statistical calculations were done using SPSS version 18 (SPSS Inc., Chicago, Illinois) and GraphPad Prism 5.03 (San Diego, California); graphs were made using GraphPad Prism 5.03.

## SUPPLEMENTARY MATERIALS FIGURES AND TABLES


