# Supplementary material for: Genetic variation in the NEIL2 DNA glycosylase gene is associated with oxidative DNA damage in BRCA2 mutation carriers
Source: Oncotarget. 2017 Nov 23;8(70):114626–36. doi: 10.18632/oncotarget.22638 (PMC5777719; doi:10.18632/oncotarget.22638)
Supplement: Supplementary file 1 [file oncotarget-08-114626-s001.pdf]

Genetic variation in the *NEIL2* DNA glycosylase gene is associated with oxidative DNA damage in *BRCA2* mutation carriers

SUPPLEMENTARY MATERIALS

Supplementary Table 1: Genetic/allelic frequencies of the *NEIL2* variant rs804271 (G>T)

	GG		GT		TT		rs804271 G>T allelic frequency
	n	%	*n*	%	*n*	%	
FBOC[*]	66	**0.39**	75	**0.45**	25	**0.15**	**0.39**
BRCA1	17	**0.42**	18	**0.45**	5	**0.13**	**0.35**
BRCA2	20	**0.43**	20	**0.43**	6	**0.13**	**0.36**
Controls	29	**0.36**	37	**0.46**	14	**0.17**	**0.4**

[*] Familial breast and ovarian cancer patients. No significant differences were detected in the genetic frequencies among FBOC group. (Pearson Chi-squared test).

Supplementary Table 2: Regulatory motifs altered by the SNP (rs804271)

Element	Ref_value	Alt_value	Match on:
			Ref: AGGGGACGGAGGCCGCATGGGCCGCCGAGCCGGGAAATCTCCGCCCCCAGCTGGAGCGG<br>Alt: AGGGGACGGAGGCCGCATGGGCCGCCGAGACGGGAAATCTCCGCCCCCAGCTGGAGCGG
E2F1	12.1	7.3	SSCGSSAAAH
SIN3A	11.2	8.4	GSNSCTSNSSNNSS
YY1	11.1	10.9	SSSNSSSSNNNSNSS

Modified from: http://archive.broadinstitute.org/mammals/haploreg/haploreg.php.

Supplementary Table 3: Gtex information summary, regarding *NEIL2* transcriptional up-regulation when rs804271 is present (30 different tissues)

Gene Symbol	SNP	Effect Size	[a]*P*-Value	Tissue
**NEIL2**	rs804271	0.52	6.80E-37	Nerve - Tibial
**NEIL2**	rs804271	0.36	5.90E-18	Heart - Atrial Appendage
**NEIL2**	rs804271	0.32	1.40E-17	Adipose - Subcutaneous
**NEIL2**	rs804271	0.31	4.50E-15	Artery - Tibial
**NEIL2**	rs804271	0.35	5.50E-15	Artery - Aorta
**NEIL2**	rs804271	0.6	1.70E-14	Ovary
**NEIL2**	rs804271	0.34	6.60E-13	Whole Blood
**NEIL2**	rs804271	0.26	2.10E-12	Thyroid
**NEIL2**	rs804271	0.24	2.60E-11	Muscle - Skeletal
**NEIL2**	rs804271	0.24	8.00E-11	Adipose - Visceral
**NEIL2**	rs804271	0.29	1.30E-10	Fibroblasts
**NEIL2**	rs804271	0.39	3.40E-09	Pituitary
**NEIL2**	rs804271	0.21	1.4E-08	Esophagus - Muscularis
**NEIL2**	rs804271	0.2	4.5E-08	Heart - Left Ventricle
**NEIL2**	rs804271	0.32	0.00000021	Colon - Sigmoid
**NEIL2**	rs804271	0.41	0.00000066	Vagina
**NEIL2**	rs804271	0.33	0.0000011	Adrenal Gland
**NEIL2**	rs804271	0.32	0.0000019	Brain - Caudate (basal ganglia)
**NEIL2**	rs804271	0.27	0.0000023	Artery - Coronary
**NEIL2**	rs804271	0.38	0.0000025	Brain - Cortex
**NEIL2**	rs804271	0.23	0.000021	Pancreas
**NEIL2**	rs804271	0.35	0.000023	Uterus
**NEIL2**	rs804271	0.19	0.000032	Stomach
**NEIL2**	rs804271	0.27	0.00028	Liver
**NEIL2**	rs804271	0.16	0.001	Lung
**NEIL2**	rs804271	0.28	0.0022	Spleen
**NEIL2**	rs804271	0.18	0.0029	Small Intestine - Terminal Ileum
**NEIL2**	rs804271	0.28	0.0033	LCLs
**NEIL2**	rs804271	0.24	0.024	Prostate
**NEIL2**	rs804271	0.09	0.026	Testis

[a]Nominal eQTL *p*-values were generated for each SNP-gene pair using a two-tailed *t* test, testing the alternative hypothesis that the beta (slope of the linear regression model) deviates from the null hypothesis of $\beta = 0$.

Supplementary Table 4: List of lymphoblastoid cell lines (LCL)

LCL [a]	BRCA1 mutationb	Mutation typec	Exon	dAge	ers804271
06S179-L[1]	Wild type	-	-	31	GT
09S797-L[2]	Wild type	-	-	27	TT
10S889-L[3]	Wild type	-	-	20	GT
11S66-L[4]	Wild type	-	-	30	GT
11S534-L[5]	Wild type	-	-	50	GT
11S954-L	Wild type	-	-	35	GT
11S375-L	Wild type	-	-	23	GT
10S1202-L	c.5123C > A; p.Ala1708Glu	Missense	18	53	GG
10S890-L[3]	c.5123C > A; p.Ala1708Glu	Missense	18	25	GT
11S65-L[6]	c.5117G > A; p.Gly1706Glug	Missense	18	31	GT
11S67-L[6]	c.5117G > A; p.Gly1706Glu	Missense	18	34	GG
07S1291-L	c.3239T > A; p.Leu1080X	Nonsense/TRC	11	34	GT
09S798-L[2]	c.2410C > T; p.Gln804X	Nonsense/TRC	11	24	GG
09S546-L	c.212 + 1G > A; p.?	Splice/TRC	5	42	TT
11S376-L[7]	c.212 + 1G > A; p.?	Splice/TRC	5	39	GT
11S384-L[7]	c.212 + 1G > A; p.?	Splice/TRC	5	75	GT
09S491-L	c.815_824dup10; p.Thr276	Frameshift/TRC	11	24	GT
10S1177-L[4]	c.68_69delAG; p.Glu23	Frameshift/TRC	2	27	TT
10S44-L	c.4309delT; p.Ser1437	Frameshift/TRC	13	22	GG
11S1004-L[5]	c.981_982delAT; p.Cys328X	Frameshift/TRC	11	25	GT

[a]1–7 LCL from relatives of the same family (sisters or mother & daughter)

[b]Mutation nomenclature based on GenBank reference sequences NM_007294.3 with numbering starting at the A of the first ATG, following the journal guidelines (www.hgvs.org/mutnomen); p.?, unknown protein nomenclature (variant causing skipping of exon 5 of BRCA1)

[c]-: Refers to the non-carrier control; TRC: Stands for truncating mutation

dAge of the woman at the time of extraction of the blood sample from which the LCL was established

[e]G>T indicate the polymorphism (rs804271).

Supplementary Table 5: Lineal regression analysis in FBOC samples: Cancer effect on the studies variables

**Independent Variable**	**Dependent variable**	**β coeff**	***p*-value**
Cancer status	*NEIL2* mRNA	-0.23	0.091
	Telomere Oxidation	0.14	0.396

We included as dependent variables *NEIL2* mRNA levels, telomere oxidation. As independent variables, we included cancer status. β coefficients quantify how much the independent variable (cancer status) modifies the dependent variables, and it shows the direction of the modification.

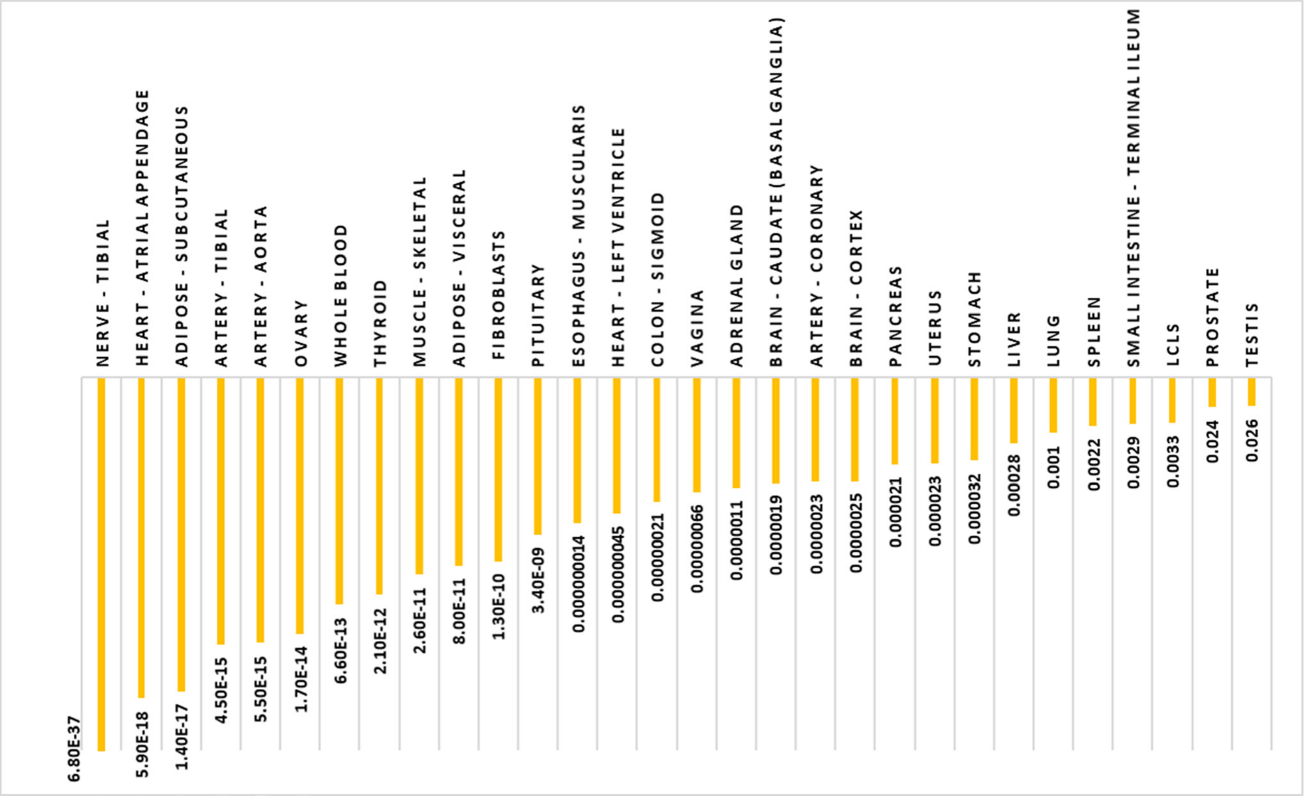

Supplementary Figure 1: Association p-values for all tissues in which the SNP has a significant effect increasing NEIL2 mRNA levels. https://www.gtexportal.org/home/snp/rs804271

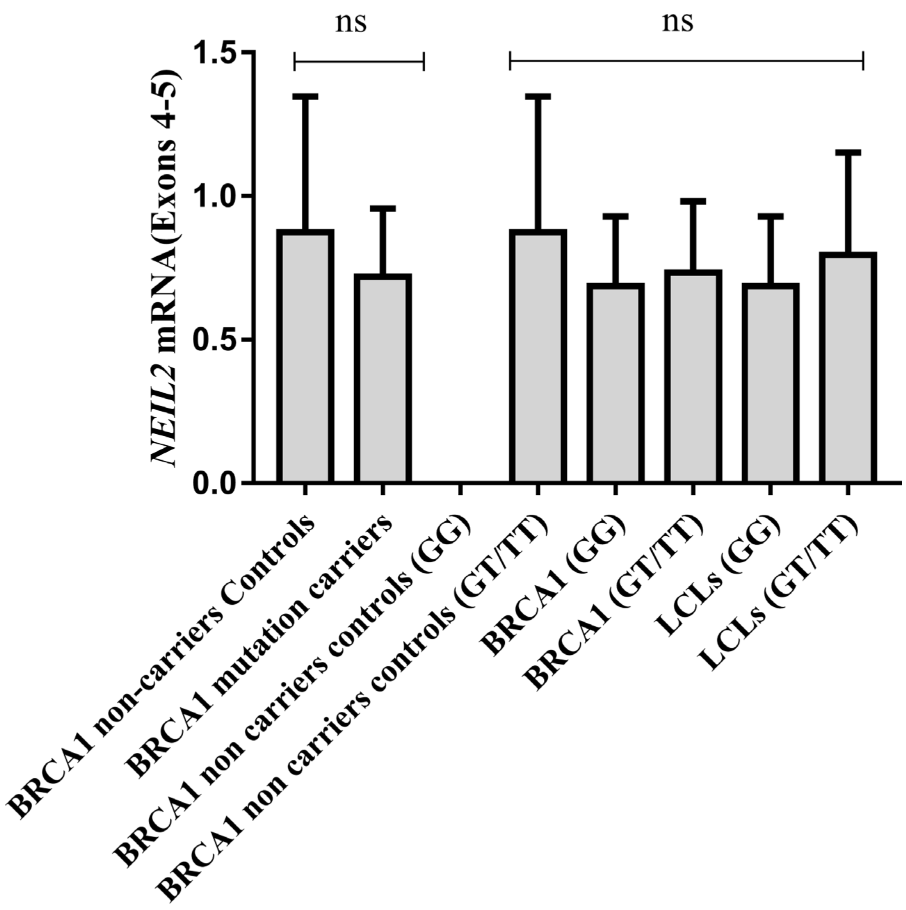

Supplementary Figure 2: Comparative analysis of NEIL2 mRNA expression according BRCA mutational status in LCLs series (BRCA1 mutation carriers are compared with non-carrier controls). Comparative analysis of NEIL2 mRNA expression according the SNP status ((carriers (GT/TT) Vs non-carriers (GG)) among LCL groups (BRCA1 mutation carriers and non-carrier controls). Bars represent the mean and the standard deviation for each group. Unpaired student t test was used to test for potential significant differences between means. No significant differences were found in any case.

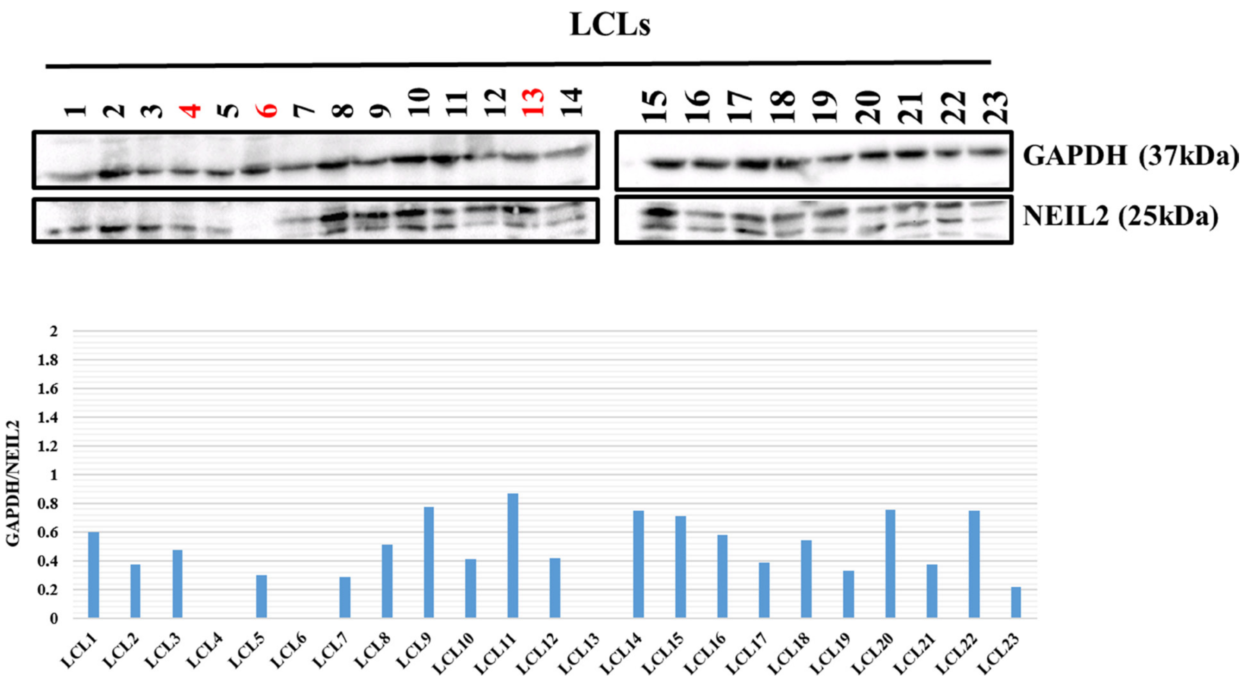

Supplementary Figure 3: NEIL2/GAPDH immunoblotting. Variation of NEIL2 protein levels (25KDa) relative to GAPDH (37kDa) as reference housekeeping gene in different LCLs (1–23). In red 3 LCLs (4,6,13) that could not be included in the final analysis.

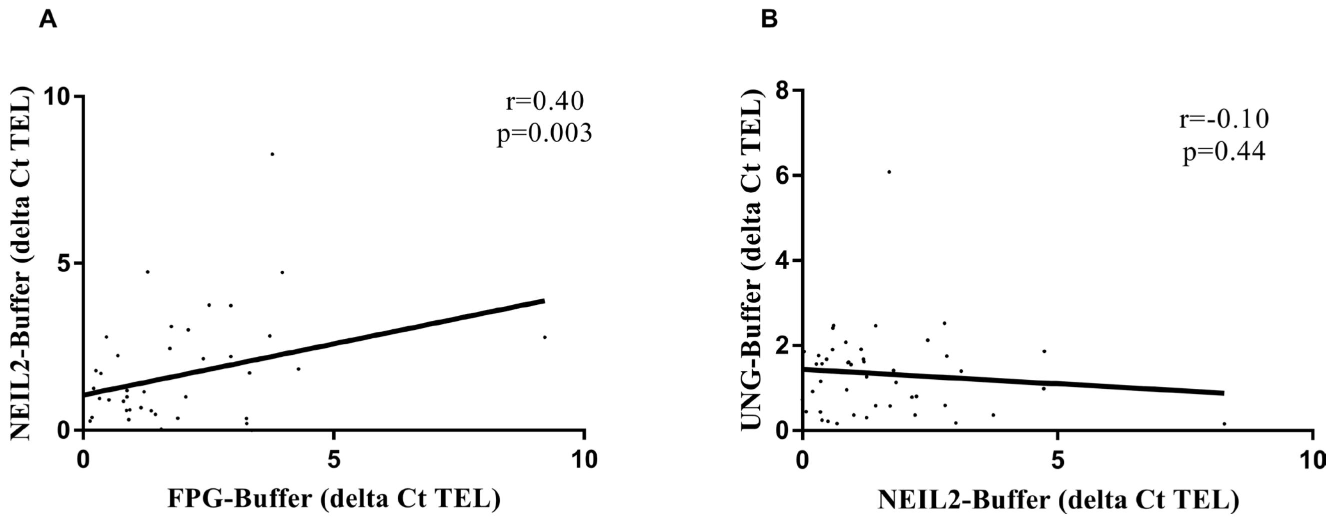

Supplementary Figure 4: (**A**) Correlation analysis between "NEIL2-lesions"and "FPG-lesions". (**B**) Correlation analysis between "UNG-lesions" and the relative amount of "uracil-lesions". Spearman test, was used to test whether correlation was significant. When *p*-value when ($p < 0.05$).

A

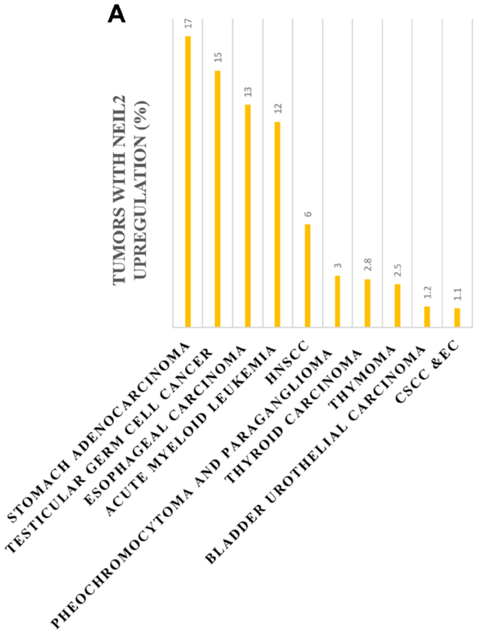

B

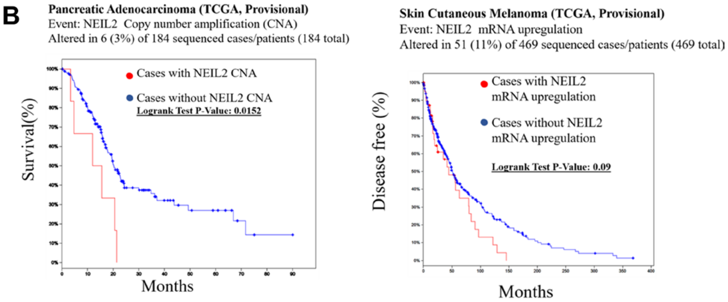

	#total cases	#cases deceased	Survival (months)
Cases with NEIL2 CNA	6	6	12.02
Cases without NEIL2 CNA	177	92	20.17

	#total cases	#cases deceased	Disease free (months)
Cases with NEIL2 mRNA upregulation	42	27	44.58
Cases without NEIL2 mRNA upregulation	360	232	51.08

Supplementary Figure 5: (**A**) Study of *NEIL2* mRNA expression among 10 different tumor types from TCGA. We selected only those studies in which *NEIL2* mRNA upregulation was mutually exclusive with *NEIL2* mRNA downregulation (none sequenced tumors). Head and Neck Squamous Cell Carcinoma (HNSCC)/ Cervical Squamous Cell Carcinoma & Endocervical Adenocarcinoma (CSCC &EC) (**B**) Two examples extracted from two independent tumor types studies from TCGA in which the event, *NEIL2* mRNA upregulation or copy number amplification, has a prognostic value in terms of overall survival (months) or disease free survival (months).
